# In-vivo liver proton density fat fraction quantification at 0.55 T: a pilot study with comparison against 3 T MRI

**DOI:** 10.1007/s10334-025-01277-9

**Published:** 2025-07-15

**Authors:** Rochelle E. Wong, Bilal Tasdelen, Ye Tian, Darryl Hwang, Sophia X. Cui, Liyun Yuan, Krishna S. Nayak

**Affiliations:** 1 Division of Gastroenterology and Hepatology, University of Southern California Keck Medical Center, 1510 San Pablo St., HC1 – Suite 200, Los Angeles, CA 90033 USA; 2 Ming Hsieh Department of Electrical and Computer Engineering, University of Southern California, Los Angeles, CA USA; 3 Department of Radiological Sciences, University of California Los Angeles, Los Angeles, CA USA; 4 Siemens Medical Solutions, Los Angeles, CA USA

**Keywords:** Liver steatosis, Proton density fat fraction, 0.55 T MRI, Low field MRI, Mid field MRI

## Abstract

**Background:**

Proton density fat fraction (PDFF)— the ratio of unconfounded fat signal to the sum of the unconfounded fat and water signals, is a valuable quantitative imaging biomarker of metabolic associated steatotic liver disease (MASLD) widely applied in clinical practice and clinical trials. PDFF of the liver is commonly measured using 3 T MRI systems. However, low-field systems are increasingly favored due to lower cost, improved safety profile, minimized artifacts around metallic implants, and enhanced patient comfort.

**Objective:**

In this pilot study, we used knowledge of standardized and widely used 3 T liver PDFF protocols, and adapted parameters to be appropriate for the 0.55 T MRI. We evaluate a liver fat quantification protocol at 0.55 T compared to a standard clinical 3 T protocol to measure liver fat in patients with MASLD.

**Material and methods:**

Eight adult patients (average age 53.6 ± 13.6 years, 5 females) with ≥ 5% PDFF on 3 T MRI underwent a 0.55 T MRI PDFF protocol within 90 days. To keep the acquisition time to be within a reasonable breath hold duration and with reasonable signal-to-noise ratio (SNR), four echoes were acquired at a lower resolution and fewer number of slices at 0.55 T compared to 3 T which uses a 6-echo multi-echo Dixon volumetric interpolated breath hold examination (VIBE) protocol. PDFF quantification accuracy of the 0.55 T approach was evaluated using a commercial PDFF phantom and in vivo.

**Results:**

In the phantom, there was excellent match (*R*^*2*^ > 0.999) between PDFF estimated by 0.55 T MRI and ground truth. Mean in vivo 3 T MRI-PDFF was 16.5%, compared to 16.3% 0.55 T MRI-PDFF (correlation coefficient *r* = 0.99). The Bland–Altman analysis showed good agreement of in vivo PDFF measurements across 0.55 T and 3 T estimating a bias or mean difference of − 0.25% and the limits of agreements (LoA) of − 3.98% and 3.48%.

**Discussion:**

Our data demonstrate that 0.55 T MRI is feasible and comparable to 3 T MRI in quantifying liver PDFF among patients with MASLD.

**Supplementary Information:**

The online version contains supplementary material available at 10.1007/s10334-025-01277-9.

## Highlights

- Evaluation of liver proton density fat fraction is feasible at 0.55T in clinically relevant populations.
- Liver proton density fat fractions using 0.55T MRI agrees well with measurements obtained from 3T MRI.
- Liver proton density fat fraction measured by 0.55T MRI shows potential as a screening and monitoring tool for patients with metabolic dysfunction-associated steatotic liver disease.

**Supplementary Information:**

The online version contains supplementary material available at 10.1007/s10334-025-01277-9.

## Introduction

In parallel with the rising obesity epidemic, metabolic-associated steatotic liver disease (MASLD), formerly known as nonalcoholic fatty liver disease, is quickly becoming the number one cause for chronic liver disease [1, 2]. Hepatic steatosis, or more commonly known as liver fat, is a hallmark of MASLD. It contributes to insulin resistance and MASLD progression, as well as cardiovascular disease [3–5]. While hepatic steatosis can be reversible with intervention, early detection and surveillance allow for timely interventions to prevent progression of metabolic-associated steatohepatitis (MASH) to liver fibrosis and cirrhosis.

The diagnosis of MASLD has gradually moved away from liver biopsy to noninvasive evaluations [6]. Ultrasound can estimate the degree of hepatic steatosis from absent, mild, moderate, to severe, but has better sensitivity when moderate-severe hepatic steatosis (> 20–30% total area of steatosis) is present [7]. Intra-operator variability, large body habitus, the presence of ascites, rib or lung shadows, and respiratory motion can also all adversely affect ultrasound results [8]. Computed tomography (CT) has high specificity for detecting hepatic steatosis, but relatively low sensitivity, especially in mild steatosis (< 20%) cases [9, 10]. Confounders such as iron or glycogen deposition, or drug therapies such as amiodarone, may affect hepatic attenuation values, and therefore fat quantification [11]. Ionizing radiation also limits the use of repeated CT exams [12].

Magnetic resonance imaging (MRI) has greatly improved the detection of steatosis. With optimized protocols, MRI can non-invasively measure even trace amounts of liver fat [13]. MRI-derived proton density fat fraction (PDFF) offers excellent sensitivity and specificity for noninvasive quantification and classification of fat, not only within the liver but also across multiple body compartments [14, 15]. This includes visceral adipose tissue, which plays a key role in insulin resistance and the pathogenesis of MASLD [16, 17]. MRI-based body composition measurement is highly sought after for MASLD surveillance and research, as it provides high-precision composition metrics not available through other modalities such as bioelectrical impedance analysis, dual X-ray absorptiometry, or CT [13]. Numerous clinical trials investigating drugs for MASLD have incorporated MRI-based body composition assessments to evaluate treatment response [18–20]. Moreover, several large longitudinal studies, including the UK Biobank and Dallas Heart Study, use MRI to quantify fat and muscle for screening and surveillance purposes [21]. MRI protocols have previously been optimized for 1.5 T and 3 T field strengths, ensuring both accuracy and reproducibility are well-established [22–25].

Recently, there has been growing interest in whole-body 0.55 T MRI systems. These systems offer several advantages, including reduced costs, improved safety profile, and reduced artifacts around air tissue interfaces and metallic implants [26]. In addition, they provide greater patient comfort, particularly for those with an obese body habitus, due to lower acoustic noise and wider bore entry points. However, 0.55 T MRI faces unique challenges, such as lower signal-to-noise ratio (SNR) due to reduced polarization, increased concomitant field effects, and diminished chemical shift resolution. Recent studies indicate that contemporary 0.55 T MRI is feasible for body applications, and sometimes favorable (e.g., high liver iron) [27–35].

In this pilot study, we proposed a new MRI liver PDFF protocol at 0.55 T MRI field strength to measure liver PDFF in patients with MASLD. We compared PDFF measurements on both field strengths, assessing the performance of 0.55 T MRI and exploring its applications in body composition metrics.

## Methods

### Patient population

Eight adult patients (age 18 +) with MASLD diagnosed by clinical hepatologists based on the diagnostic criteria of the American Association for the Study of Liver Diseases (AASLD) guideline [36], presented for standard 3 T abdominal MRI and novel 0.55 T MRI PDFF protocol within 90 days of each other.

### Prospective study design

Patients were recruited to this prospective pilot study if they met the above-mentioned inclusion criteria. The 0.55 T MRI was then performed within 90 days of 3 T MRI to minimize confounding variables. Some possible confounders included interval change in weight between 3 T and 0.55 T MRI scans, and interval change in hepatic function panel lab values or hepatic inflammation between 3 T and 0.55 T MRI scans. This study was approved by the University of Southern California Institutional Review Board (IRB), Approval ID: HS-22-00705, with written and informed consent obtained from each subject.

### Imaging methods

A multi-echo three-dimensional (3D) gradient echo (volumetric interpolated breath hold examination — VIBE) pulse sequence was used for data acquisition. A multi-step approach with Dixon fat–water separation and confounder-corrected nonlinear fitting was used for fat quantification [37]. At both 3 T and 0.55 T, multi-echo Dixon VIBE was acquired in the axial orientation, centered on the liver, and during a single breath hold.

3 T imaging was performed on a whole-body scanner (MAGNETOM Vida, Siemens Healthineers, Forchheim, Germany) equipped with the vendors’ commercial product option for online PDFF map reconstruction (LiverLab). The vendor provided multi-echo Dixon VIBE protocol for PDFF quantification (qDixon) was used. Imaging parameters include repetition time (TR) = 9 ms, flip angle = 4°, matrix size = 160 × 111, slice thickness = 3.5 mm, bandwidth = 1080 Hz/Pixel. Six echoes were acquired with an acceleration factor of 4 leading to an acquisition time of 13 s. An 18-channel flexible body array and a 32-channel spine array were used for signal reception. A list of scan parameters is summarized in Table 1.

**Table 1:** Representative imaging parameters for 3 T and 0.55 T acquisitions

Parameter	3 T	0.55 T
FOV (mm^2^)	380 × 333	450 × 394
Matrix size	160 × 111	128 × 73
Partial Fourier	Off	Phase 7/8, slice 6/8
Slice thickness (mm)	3.5	3.5
Number of Slices	64	52
TR (ms)	9	19
TE (ms)	1.05, 2.46, 3.69, 4.92, 6.15, 7.38	2.43, 6.53, 10.63, 14.73
Number of echoes	6	4
Bandwidth (Hz/pixels)	1080	250
Flip angle (degrees)	4	6
CAIPIRINHA acceleration factor	4	2
Acquisition time (s)	13	18

0.55 T MRI was performed on a prototype whole-body system with high-performance gradients (prototype MAGNETOM Aera, Siemens Healthineers, Forchheim, Germany), which is ramped down from 1.5 T [27]. A 6-channel flexible body array and an 18-channel spine array were used for signal reception. At 0.55 T, the phase cycling of fat and water between in-phase and out-of-phase conditions is approximately 6 times slower than at 3 T. Therefore, in order to keep the acquisition time to be within a reasonable breath hold duration, four echoes were acquired at echo times (TE) of 2.43 ms, 6.53 ms, 10.63 ms, and 14.73 ms, as opposed to the 6 echoes acquired at 3 T. The corresponding TR was 19 ms. Similarly, a smaller number of slices were acquired at 0.55 T at 42 slices, compared to 64 slices acquired at 3 T to limit scan time to be within one breath-hold. Imaging parameters were adapted to compensate for the reduced SNR at 0.55 T compared to higher field strength. These parameters include a smaller matrix size of 128 × 73, larger field of view (FOV) of 450 × 394 mm^2^, lower bandwidth of 250 Hz/Pixel, and a lower acceleration factor of 2. Flip-angle was set to 6° to provide a good compromise between low T1-bias for quantification while maintaining an adequate SNR. The acquisition time was 18 s.

PDFF quantification accuracy of the 0.55 T approach was evaluated using a PDFF phantom (Model 300, Calimetrix, Madison, WI) [38, 39]. PDFF values were measured for all 12 vials using manually placed cylindrical ROIs with a radius of 8.8 mm and 24 mm height across the slice direction. Mean and standard deviations of the PDFF values were calculated within each vial. The measured PDFF values were compared against the ground-truth PDFF values provided by the phantom manufacturer, which is measured at a 3 T field-strength [38].

PDFF calculation from the 0.55 T acquisition was performed using the prototype LiverLab PDFF algorithm for the phantom experiment, and by an offline research implementation of the LiverLab PDFF algorithm for the in-vivo experiments. For both, the fat/water chemical shifts were scaled according to the ratio of field strengths.

### Image analysis

For each subject, 3 T MRI and 0.55 T MRI images were assessed by a radiologist who was blinded to the study subject. Liver fat quantification was reported using PDFF, a ratio of unconfounded fat signal to the sum of the unconfounded fat and water signals. Liver volumes were segmented using an automated software, TotalSegmentator [40]. After the automatic segmentation, volumes were eroded by three pixels using binary erosion. Liver PDFF values were then calculated from the eroded volumes to avoid boundary effects. Liver PDFF agreement between 3 T and 0.55 T MRI was then evaluated using linear correlation and Bland–Altman analysis.

## Results

### Phantom PDFF results

**Fig. 1:**
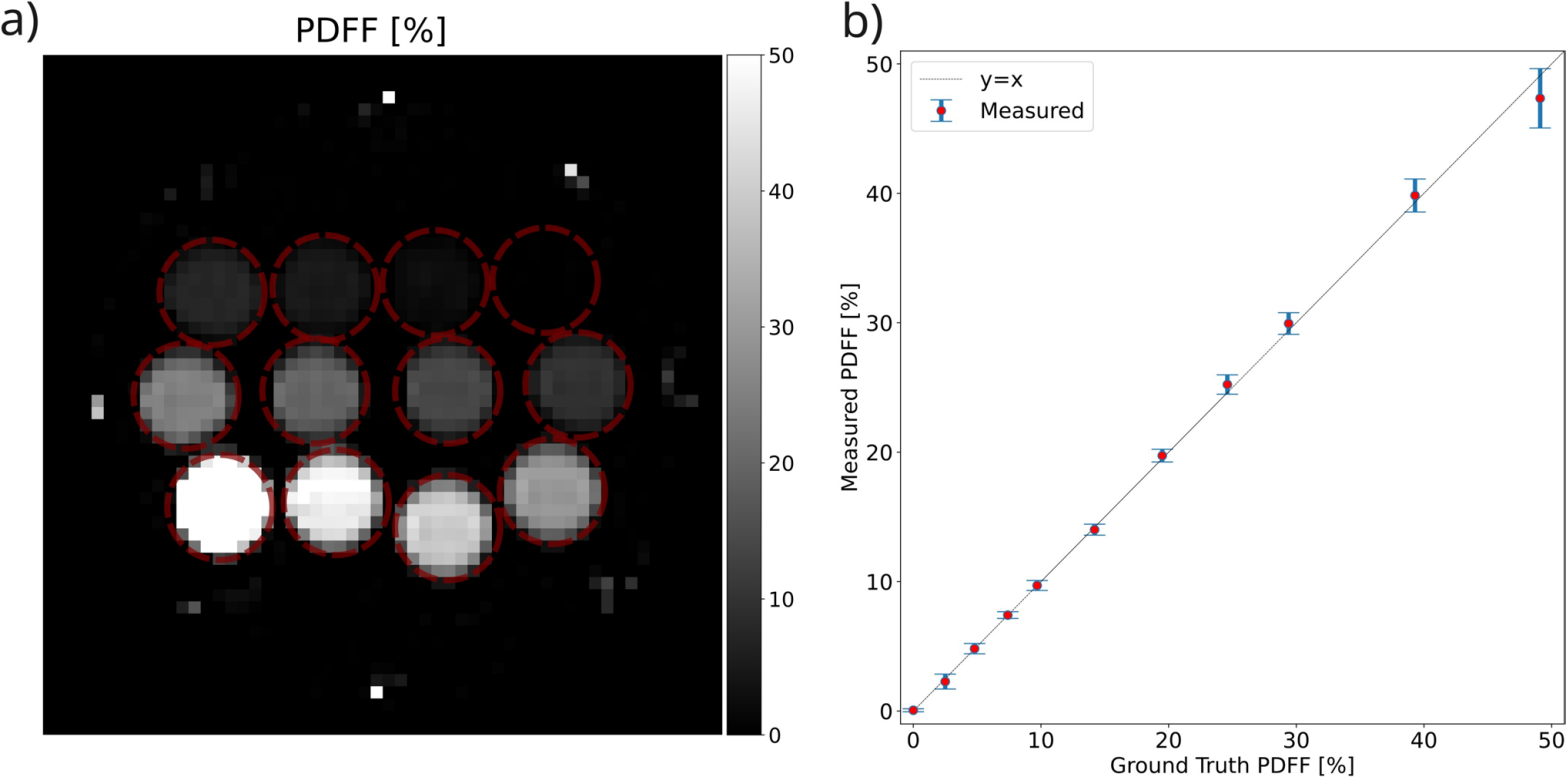
PDFF quantification accuracy validation using Calimetrix Model 300 phantom. **a** Mid-slice of the measured PDFF maps and **b** comparison of measured PDFF values and ground truth PDFF values. Error bars show the standard deviation of the measurements within each vial. Note that for both (**a**) and (**b**), the 100% vial is excluded to focus the values on the clinically relevant range

As shown in Fig. 1, we observed excellent match (*R*^*2*^ > 0.999) between ground truth PDFF and mean PDFF estimated by 0.55 T MRI.

### In-vivo PDFF results

**Fig. 2:**
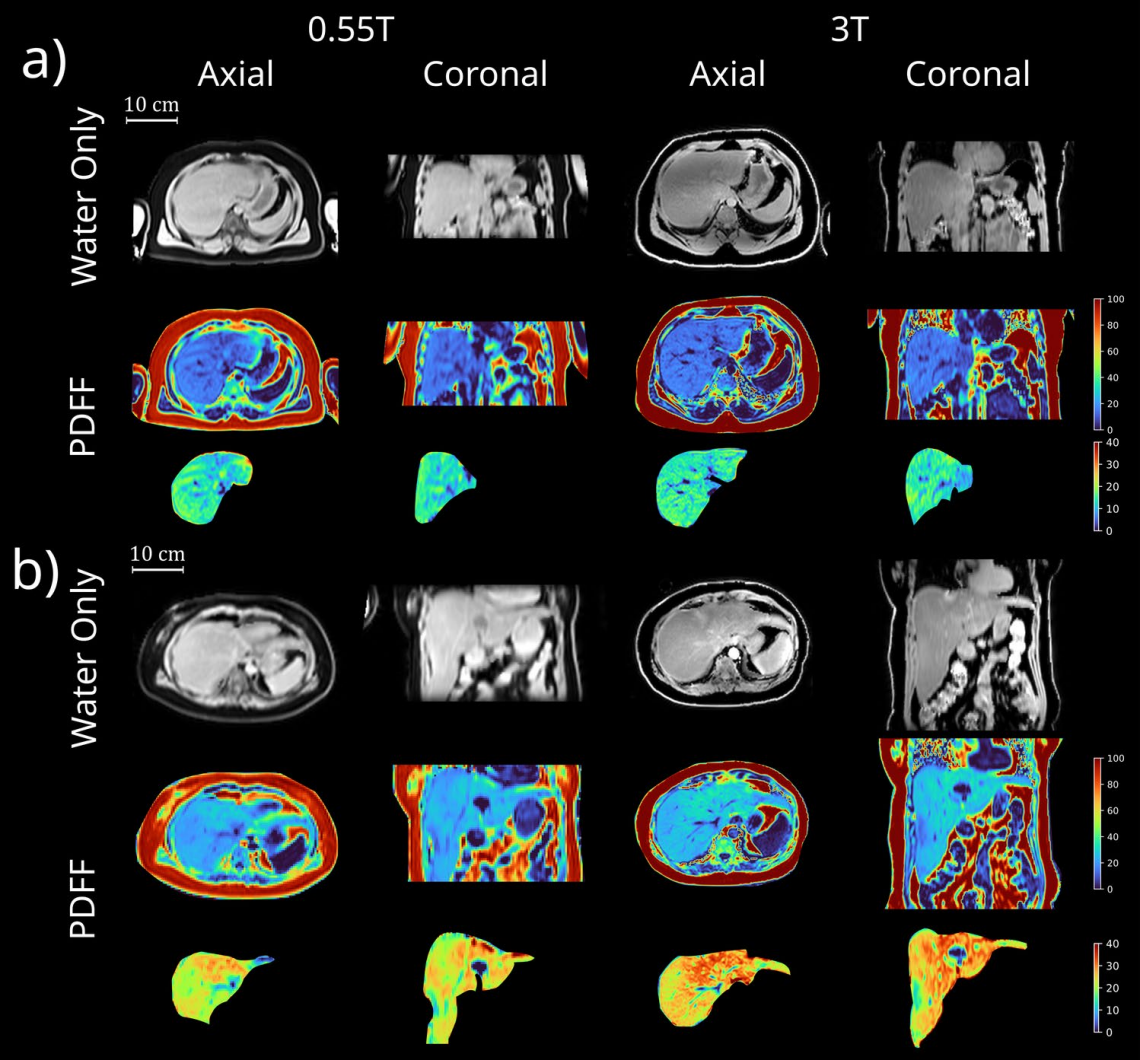
Representative 0.55 T and 3 T image quality from two subjects. Shown are axial and coronal images from the water only images and PDFF maps. **a** Age 37, male, BMI = 32.9 kg/m^2^, 3T PDFF = 13.93%, 0.55T PDFF = 13.73%; **b** Age 55, female, BMI = 25.4 kg/m^2^, 3T PDFF = 24.81%, 0.55T PDFF = 25.2%

Eight patients (age 53.6 ± 13.6 years, 5 females) were included in this study and underwent both 3 T and 0.55 T MRI. 0.55 T MRI imaging was successful in all 8 subjects, with representative image quality shown in Fig. 2.

**Fig. 3:**
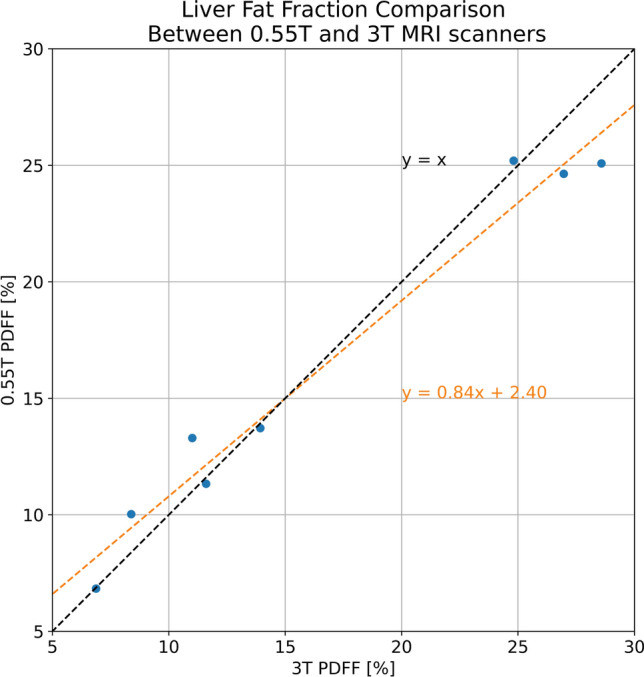
Comparison of Liver Fat Fraction Calculated From 3 T and novel 0.55 T MRI. Orange dashed line shows the slope and intercept of the line fit. Black dashed line shows the y = x line

**Fig. 4:**
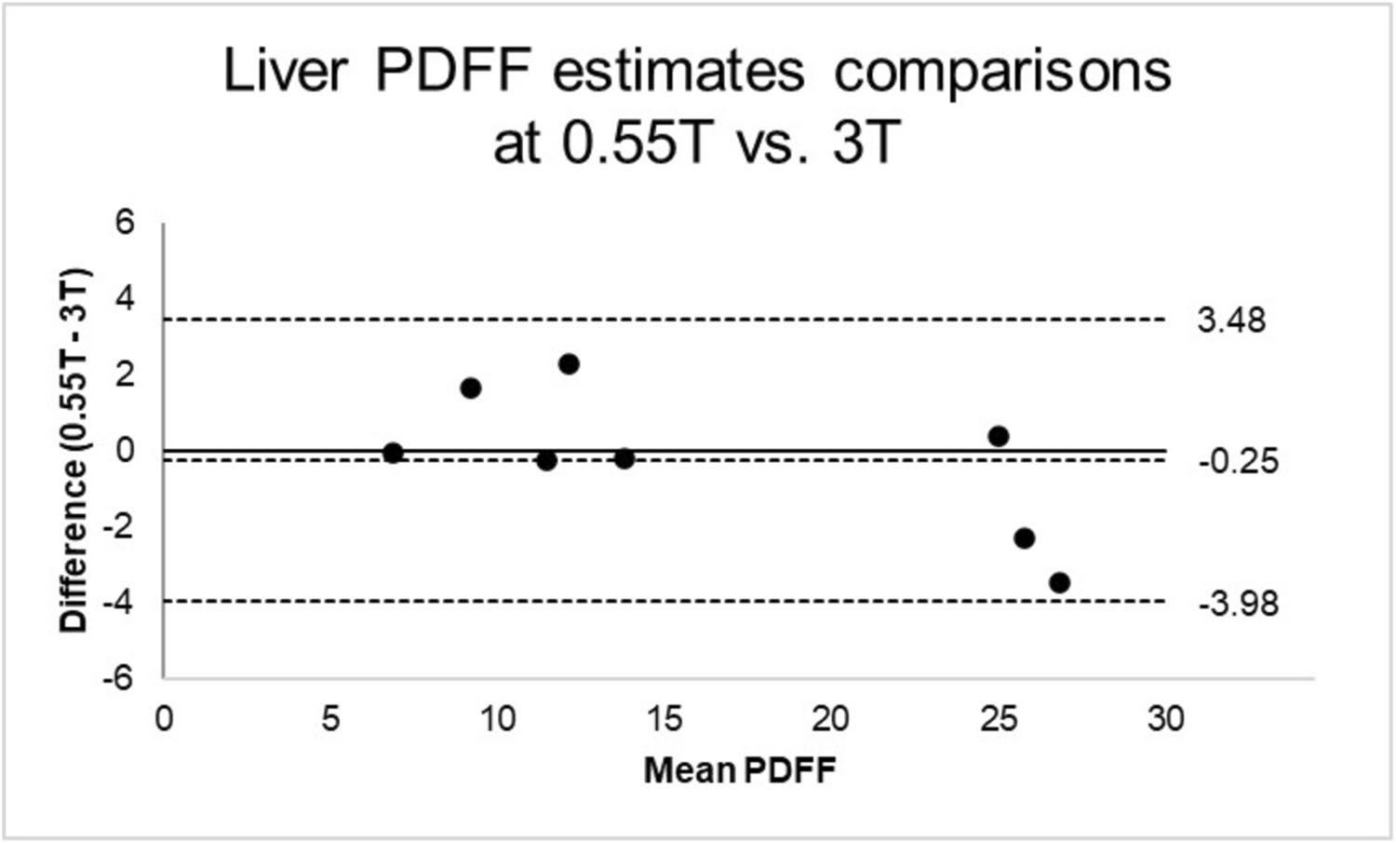
Bland–Altman plot comparing the PDFF estimates from the 0.55 T and 3 T acquisitions. The central horizontal dashed line represents the mean difference or bias of -0.25%, with lower and upper dashed lines representing the limits of agreements (LoA) of -3.98% and 3.48%

Mean liver fat measured with 0.55 T MRI is 16.3% ± 7.5%, comparable to 16.5% ± 8.8% measured with 3 T MRI with correlation coefficient *r* = 0.99, as shown in Fig. 3. Correlation analysis performed showed strong positive correlation. It is reproducible across scanner protocol and field strength. The Bland–Altman plot in Fig. 4 shows good agreement of PDFF measurements across 0.55 T and 3 T. The bias or mean difference between the PDFF measured 0.55 T vs. 3 T was − 0.25% (central horizontal dashed line) and the limits of agreements (LoA) were of -3.98% and 3.48%.

### Patient demographics

The presenting comorbidities of this cohort included prediabetes/diabetes (40%), hypertension (60%), and hyperlipidemia (50%). Mean aspartate transaminase (AST) was 52.2 ± 32.9 U/L and mean alanine transaminase (ALT) was 69.9 ± 53.5 U/L. Metabolic markers included mean low-density lipoprotein (LDL) 96.5 ± 35.6 mg/dL, high-density lipoprotein (HDL) 42.3 ± 7.9 mg/dL, total cholesterol 178.3 mg/dL ± 31.6, and hemoglobin A1c 6.7 ± 1.4. Mean fibrosis-4 (FIB4) score was 1.9 ± 1.4. Mean liver stiffness measurement as measured by liver elastography was 7.4 ± 3.0 kPa. Demographics can be found in Supporting Information Table 1. Mean time interval between 3 T and novel 0.55 T MRI was 28.1 ± 20.7 days.

## Discussion

Overall, liver PDFF quantification agreed well between standard 3 T and novel 0.55 T MRI protocols. A strength of this study includes the large range of PDFF values represented across a wide range of steatosis and fibrosis stages, despite the small cohort of subjects. Fibrosis stages ranged from minimal fibrosis F0 to advanced fibrosis/cirrhosis F4, while PDFF ranged from 6.8% to 28.5% PDFF. Many of the patients also had other modalities to corroborate their degree of steatosis seen on MRI, including Fibroscan and liver biopsy. Larger differences in fat fraction were seen at higher degrees of steatosis, which may be due to T1 bias, noise bias, and/or an insufficient number of echoes (at 0.55 T) in this initial study. Hepatic fibrosis is known to elongate lean T1 [41], which could make T1 bias worse. High flip angle and short TR can cause the fat signal to appear artificially stronger compared to the water signal. The proposed protocol could be augmented with a T1 mapping sequence to further investigate the role of elongated T1 with liver scarring and the possible link of PDFF bias to higher scarring. Carefully balancing flip angle and TR is key to accurately assess PDFF while minimizing noise. Lower field strength MRI may be able to tolerate higher flip angle and tolerate more T1 bias. This will require further protocol optimization to minimize discrepancies.

This study has limitations. First, the two MRI acquisitions were not performed on the same day, which may have introduced confounding variables based on time of day, scan location, interval weight changes and serum lab fluctuations. We limited the interval of two MRIs within 90 days and the subjects had no more than 5% body weight change over this interval. Second, we made our best efforts to experimentally optimize the 0.55 T protocol, however there is room for further optimization. In contrast to 6 echoes used for 3 T, only 4 echoes were used at 0.55 T, due to the longer inter-echo spacing. This number of echoes may be suboptimal for fat quantification [42]. Furthermore, to reduce T1 bias, flip angle was kept relatively small, however, the resulting noise bias could become confounding factors. Third, we observed a discrepancy between quantified liver volumes for some subjects. This stemmed from the use of an insufficient number of slices at 0.55 T, which failed to cover the entire liver in some subjects, specifically those that had the largest extent in the superior-inferior direction. Figure 1 illustrates the difference in superior-inferior coverage. In hindsight, an increase in slice thickness at 0.55 T from 3.5 mm to 4 mm or 4.5 mm would have been appropriate to preserve the S-I coverage. Fourth, there are known challenges with using phantoms for evaluation of PDFF biomarker accuracy. There are potential issues with temperature dependence [43] and with matching of fat and lean T1 values which are field strength dependent [44, 45]. The phantom used in this study has been primarily validated at 1.5 T and 3 T, and its performance match for 0.55 T has not yet been comprehensively studied.

This novel 0.55 T MRI fat quantification approach has potential for many future translational applications. It could be used to screen the general population for high metabolic risk profiles at high risk for developing steatotic liver disease. It can be used to monitor allograft health in post-liver transplant patients, or to optimize the quality of pre-transplant donated livers, especially with the increasing use of living donor liver transplant. In day-to-day application, this MRI can be used to noninvasively diagnose MASLD and monitor treatment response and fat distribution, with potential for tissue-targeted adipose reduction therapy in future. Further research is needed to establish scan-to-scan repeatability as well as improve cross-field-strength reproducibility and evaluate patient satisfaction for this potential new imaging biomarker as a promising alternative to liver biopsy.

## Conclusion

To our knowledge, this is the first study to demonstrate the use of 0.55 T MRI for liver fat quantification in a clinically relevant population. Our results demonstrate that 0.55 T MRI is feasible and well-correlated to 3 T MRI in quantifying liver fat. Lower field strength may be favorable for this application because of reduced costs, improved safety profile, reduced artifact around air-tissue interfaces and metallic implants, and because it is more tolerable to patients due to reduced acoustic noise and wider bore entry points. The liver PDFF imaging biomarker may be a promising alternative to liver biopsy to diagnose and monitor MASLD. Further research is needed to assess patient tolerability. Further research is also needed into the root cause of the observed cross-field-strength bias, and how it can be improved.

## Supplementary Information

Below is the link to the electronic supplementary material.

Supplementary file1 (DOCX 31 KB)

## Data Availability

The datasets used and analyzed in this current study are available from the corresponding author upon reasonable request.

## Abbreviations

ALT: Alanine transaminase
AST: Aspartate transaminase
FIB4: Fibrosis-4 score
FOV: Field of view
HDL: High density lipoprotein
LoA: Limits of agreement
LDL: Low density lipoprotein
MASH: Metabolic associated steatohepatitis
MASLD: Metabolic associated steatotic liver disease
MRI: Magnetic resonance imaging
PDFF: Proton density fat fraction
SNR: Signal to noise ratio
TE: Echo time
TR: Repetition time
VIBE: Volumetric interpolated breath hold examination
