# Supplementary material for: In-vivo liver proton density fat fraction quantification at 0.55 T: a pilot study with comparison against 3 T MRI
Source: MAGMA. 2025 Jul 15;38(6):949–57. doi: 10.1007/s10334-025-01277-9 (PMC12638424; doi:10.1007/s10334-025-01277-9)
Supplement: Supplementary file 1 — Supplementary file1 (DOCX 31 KB) [file 10334_2025_1277_MOESM1_ESM.docx]

**Supporting Information Tables**

**Supporting Information Table 1. Cohort Demographics**

| **Demographics (n=8)** | | | **Mean** | **Std** |  |
| --- | --- | --- | --- | --- | --- |
| Age (years) | | | 53.6 | 13.6 |  |
| Sex (female) | | | 5 (63%) |  |  |
| BMI |  | | 31.2 | 7.7 |  |
| **Ethnicity** |  | | **n(%)** |  |  |
|  | Non-Hispanic White | | 2 (25%) |  |  |
|  | Hispanic/Latinx | | 2 (25%) |  |  |
|  | Non-Hispanic Black | | 1 (12.5%) |  |  |
|  | Asian/Pacific Islander | | 2 (20%) |  |  |
|  | Other | | 1 (12.5%) |  |  |
| **Metabolic Syndrome** | | | **n(%)** |  |  |
|  | Hypertension (HTN) | | 6 (75%) |  |  |
|  | Prediabetes/Diabetes | | 4 (50%) |  |  |
|  | Hyperlipidemia (HLD) | | 5 (63%) |  |  |
| **Labs** |  | | **Mean** | **Std** | **Normal Range Values** |
|  | Aspartate transaminase (AST) (U/L) | | 52.2 | 32.9 | 10-35 |
|  | Alanine transaminase (ALT) (U/L) | | 69.9 | 53.5 | 10-35 |
|  | Total bilirubin (mg/dL) | | 0.5 | 0.2 | 0.2-1.0 |
|  | Alkaline Phosphatase (IU/L) | | 240.8 | 443.2 | 44-145 |
|  | Albumin (g/dL) | | 4.4 | 0.2 | 3.5-5.5 |
|  | International Normalized ratio (INR) | | 1 | 0 | 0.8-1.2 |
|  | Platelets (PLT/mcL) | | 264.9 | 155 | 150-450 |
|  | Low-density lipoprotein (LDL) (mg/dL) | | 96.5 | 35.6 | <100 |
|  | High-density lipoprotein (HDL) (mg/dL) | | 42.3 | 7.9 | 40-59 |
|  | Triglycerides (mg/dL) | | 197.9 | 130.1 | <150 |
|  | Total cholesterol (mg/dL) | | 178.3 | 31.6 | <200 |
|  | Fasting glucose* (mg/dL) | | 118 | 37.3 | 70-100 |
|  | Fasting insulin^ (mIU/L) | | 17.4 | 4.2 | <25 |
|  | Hemoglobin A1c (HbA1c) (%) | | 6.7 | 1.6 | <5.7 |
|  | Fibrosis-4 (FIB-4) Score | | 1.9 | 1.4 | <1.30 |
| **Fibroscan** | |  | **Mean** | **Std** |  |
|  | Liver stiffness measurement (kPa)* | | 7.4 | 3 | 2-7 |
|  | Controlled attenuation parameter (CAP) score (dB/m)^ | | 318 | 45 | <236 |
| **Liver Biopsy Fibrosis Stage (n=5)** | | | **n(%)** |  |  |
|  | 0 | | 1 (20%) |  |  |
|  | 1 | | 1 (20%) |  |  |
|  | 2 | | 0 (0%) |  |  |
|  | 3 | | 1 (20%) |  |  |
|  | 4 | | 2 (40%) |  |  |

*data available only for 7 patients

^data available only for 5 patients
